# Qualitative Characteristics and Functional Properties of Cherry Tomato under Soilless Culture Depending on Rootstock Variety, Harvesting Time and Bunch Portion

**DOI:** 10.3390/foods13101450

**Published:** 2024-05-08

**Authors:** Anna Rita Rivelli, Donato Castronuovo, Barbara La Gatta, Maria Teresa Liberatore, Angela Libutti

**Affiliations:** 1 School of Agricultural, Forest, Food and Environmental Sciences, University of Basilicata, Via dell’Ateneo Lucano, 10, 85100 Potenza, Italy; annarita.rivelli@unibas.it (A.R.R.); donato.castronuovo@unibas.it (D.C.); 2 Department of Agricultural Sciences, Food, Natural Resources and Engineering (DAFNE), University of Foggia, Via Napoli, 25, 71122 Foggia, Italy; barbara.lagatta@unifg.it (B.L.G.); mariateresa.liberatore@unifg.it (M.T.L.)

**Keywords:** *Solanum lycopersicum* L., greenhouse tomato cultivation, grafted cherry tomato, marketable fruit yield, polyphenols, lycopene, β-carotene

## Abstract

Tomato grafting is an effective practice in increasing the profitability of fresh-market tomato cultivation, especially in greenhouses, and is also considered a strategy for enhancing fruit quality. In this study, selected quanti-qualitative traits, and the of bioactive health-promoting compound and organic acid contents of cherry tomato fruits from three different scion/rootstock combinations (Sunstream/Top Bental, Sunstream/Kaiser and Sunstream/Suzuka) grown under a greenhouse hydroponic system were evaluated in three different harvests (beginning, middle and end of the whole harvesting period) and on three different bunch portions (proximal, central and distal). Although the tomato productive performance was influenced by the rootstock, with Suzuka and Kaiser grafted plants showing the highest total marketable yield (9.8 kg plant^−1^, i.e., 20% more than Top Bental), the yield-related traits (bunch number, weight and length per plant, and fruit number per bunch) and the qualitative characteristics of the fruits (color, equatorial and polar diameters, dry matter and solid soluble contents, pH and titratable acidity) showed less variability, by displaying, along with the bioactive compound contents (total polyphenols, lycopene, β-carotene), DPPH free radical scavenging activity and organic acids contents (lactic and acetic), a significant effect of the harvesting time and bunch portion. Fruits from the beginning of the harvesting period showed better qualitative and functional properties, with the lycopene and β-carotene contents equal to 178.6 and 3 mg 100 g^−1^ fw, and fruits from proximal and central bunch portions had lycopene and β-carotene contents equal to 203.1 and 2.9 mg 100 g^−1^ fw.

## 1. Introduction

Tomato is the most cultivated horticultural crop with a global production of 186.11 million tons year^−1^ and a harvested area of 4.92 million ha [1]. China is the largest producer, accounting for 36% of the global production, followed by India (11%), Turkey (7%) and the United States of America (6%). Italy is in fifth place and is the highest producer in Europe, accounting for 6.14 million tons year^−1^ of tomato fruits produced on 97,610 ha [1]. However, several abiotic and biotic stresses pose a significant challenge to global tomato production. Indeed, the growth and yield of this crop can be affected by water availability, temperature and salinity, as well as by a number of plant diseases caused by fungus, bacteria, viruses, nematodes and pests [2,3]. In recent years, tomato grafting has emerged as a potential tool to reduce plant susceptibility to suboptimal growth conditions and achieve better overall crop performance [4]. Initially proposed just to control soil-borne pathogens [5], grafting soon became an agronomic practice used to enhance tomato tolerance to drought, high-saline conditions, low and high temperatures [6,7,8,9] and soil contamination by heavy metals [10,11]; it was also used to improve plant nutrient uptake and water use efficiency [12]. Interestingly, previous studies report the possibility of using tomato grafting to increase the crop yield and the quality of fruits [13,14]. Nowadays, the use of grafted tomato seedlings is an environmentally friendly, sustainable and relatively low-cost practice [15] and is considered a standard in the greenhouse production of fresh-market tomatoes [16].

Tomato fruits are extensively used both fresh and cooked with an average daily intake per capita that is higher than other vegetables. They are a main component of the traditional Mediterranean diet [17] and are a rich source of nutrients and bioactive compounds that promote human health and wellness [18]. The high concentration of biologically active molecules that can detoxify reactive oxygen species and prevent oxidative changes in the human body is associated with a lower risk of cancer and the prevention of cardiovascular and neurodegenerative diseases [2]. Carotenoids (lycopene and β-carotene), flavonoids (quercetin, kaempferol, myricetin, naringenin, rutin and lutein), phenolic acids (caffeic, ferulic, p-cumaric and chlorogenic acids), ascorbic acid (vitamin C) and tocopherol (vitamin E) are the major contributors to the high antioxidant capacity of tomato fruits [19]. Lycopene, which represents 80% of the total carotenoid content, is one of the most powerful antioxidants and its intake has been correlated with lower incidences of gastrointestinal, stomach and prostate cancers [20], and antidiabetic, anti-atherogenic, antithrombotic, antimicrobial, anti-allergenic and anti-inflammatory effects [21,22,23]. Lycopene is also responsible for the red color of ripe fruits, one of the main quality attributes required by the industry and consumers. β-carotene constitutes 7% of the total carotenoid content and has strong chemoprotective functions and high provitamin A activity. Moreover, tomato is a good source of essential elements (Ca, K, Na, P, Mg, S, Cl, Fe, Cu, I, Zn, etc.), protein, sugar, fatty acids, cellulose, pectin and organic acids [19]. The sugar and organic acid content, responsible for the sweet and sour flavors, respectively, affect the tomato taste, an important parameter for the organoleptic fruit quality.

Among cultivated fresh-market tomato types, cherry tomatoes are highly appreciated by consumers for their small size, sweet taste and easy consumption in snack, salads or other dishes. Compared with large-sized fruits, they are rich in sugars (glucose and fructose) and organic acids (citric and malic acids), which lead to a more intense flavor; they are also reach in organic volatile compounds, which determine a more intense aroma [24], lycopene, β-carotene and other nutrients [25]. This tomato variety is particularly suitable for soilless cultivation in greenhouses, which has high yield and quality standards, allowing for production to occur all year round. Several studies report that the yield, the qualitative characteristics, and the nutritional and functional traits of cherry tomatoes are influenced by a number of pre-harvest and post-harvest factors, including genotype, environmental conditions, cultivation practices, grafting, rootstock type, fruit position on the bunch, harvesting period, ripening stage at harvest, storage conditions and duration, and post-harvest treatments [4,26,27,28,29,30,31,32,33,34,35,36].

Therefore, the aim of the present study was to test the cherry tomato cultivar Sunstream, grafted onto three different hybrid tomato rootstock varieties, i.e., Top Bental, Kaiser and Suzuka, and grown in soilless culture in a greenhouse, in order to evaluate the yield-related traits of the plants, and the qualitative characteristics and the functional properties of the fruits depending on the rootstock type, harvesting time and bunch portion.

## 2. Materials and Methods

### 2.1. Experimental Design and Cultivation Conditions

**Figure 1:**
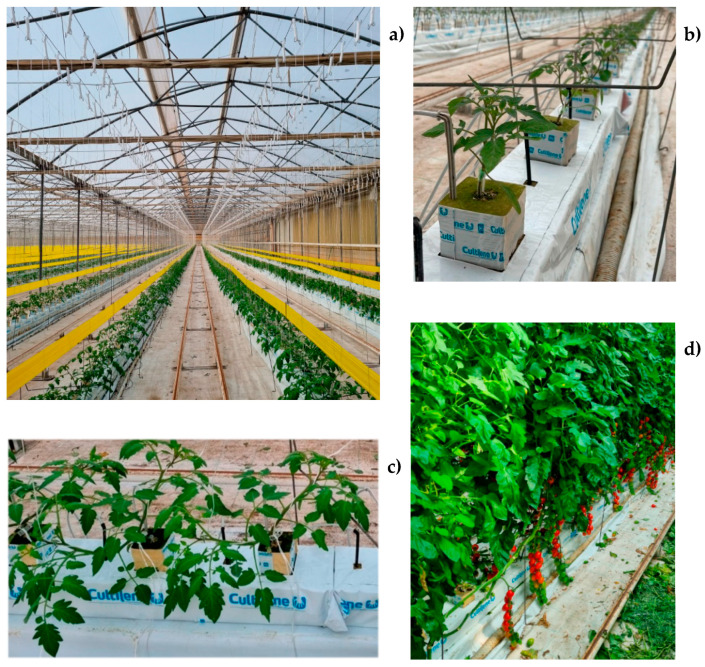
Overview of grafted cherry tomato plants grown under a hydroponic system in the greenhouse of “Ortoserre” farm after transplanting (**a**); seedlings just after transplantation on stone wool slabs (**b**); growing plants (**c**); plants during the productive period (**d**).

The trial was carried out during the 2022 season (March–October) using cherry tomato (*Solanum lycopersicum* L. var. *cerasiforme*) grown in a hydroponic system on stone wool slabs in the greenhouse at “Ortoserre” farm (41°04′57″ N, 15°40′29″ E, 185 m a.s.l.) (Figure 1). The farm is located in the Ofanto Plain, Southern Italy, an agricultural area particularly dedicated to tomato cultivation in both protected and open field conditions. The greenhouse (150 m wide and 201 m long) extended over a 3-hectare area and was subdivided into 15 spans (10 m wide and 201 m long) and consisted of a metal structure covered with a 200 μm diffusive plastic film (C/497 Sunsaver, Polyeur S.r.l., Benevento, Italy). It was equipped with an automated roof opening and shading device and an automated heating system and had a 4.3 m^3^ m^−2^ volume/surface area index.

For the experiment, cherry tomato plants grafted onto three specific tomato rootstock varieties were used. In particular, the scion was the cherry tomato cultivar Sunstream (S) (Enza Zaden, Italia S.r.l., Tarquinia, Viterbo, Italy) and the tested rootstocks were the three commercial hybrids Top Bental (TB) (TSI Italia S.r.l., Foggia, Italy), Suzuka (Su) and Kaiser (Ka) (Rijk Zwaan Italia S.r.l., Bologna, Italy). The characteristics of vigor and genetic resistance to soil-borne pathogens of the rootstock varieties are reported in Table 1. The scion/rootstock combinations Sunstream/Top Bental (S/TB), Sunstream/Suzuka (S/Su) and Sunstream/Kaiser (S/Ka) were the three experimental treatments that were compared, which were arranged according to a randomized complete block design with three replicates. Each replicate consisted of 2 plants for a total of 6 plants per treatment and 18 plants included in the experiment. In particular, the S/TB treatment was considered as the control since the host farm uses this scion/rootstock combination as a standard for cherry tomato cultivation. The use of grafted plants is now a common practice adopted by the host farm to increase the quali-quantitative characteristics and realize a positive return on farm investment.

The grafted tomato seedlings, provided by a specialized nursery, were preventively placed in stone wool cubes (Pc, Saint-Gobain Cutilene B.V., Rijen, The Netherlands) that were 75 mm long, 75 mm wide and 50 mm high with a single hole that was 22 mm in diameter and 27 mm in depth to allow for complete rooting. On March 18th, the seedlings at the 3rd–4th true leaf stage were transplanted on stone wool slabs (MaXXima, Saint-Gobain Cutilene B.V., Rijen, The Netherlands) that were 120 cm long, 15 cm wide and 10 cm high. Slabs with holes 30 cm apart were placed in rows 200 cm apart in order to have a density of 1.6 plants m^−2^. The cultivation of the cherry tomato plants started with a density of 3.2 productive stems m^−2^ (2 stems plant^−1^), which increased to 4.8 productive stems m^−2^ on day 61 after transplanting (DAT) and to 6.4 productive stems m^−2^ on 116 DAT by keeping three and four stems per plant, respectively. As growth proceeded, the tomato plants were tutored using vertical wires and a regular lateral shoot removal was performed every 4–5 days. The plants were managed according to the host farm’s standard practices. In particular, they were fertigated daily with a nutrient solution whose macro- and micro-element composition varied according to the growth stage, through an automatically controlled drip irrigation system, which ensured the optimal water and nutrient supply to each plant. The reproductive phase of the crop cycle started on 34 DAT when plants began to produce the first flowers. Flower pollination was performed by bumblebees (*Bombus terrestris* L.) whose hives were located throughout the greenhouse.

The harvesting period started on 82 DAT and ended on 215 DAT. Tomato bunches were hand-picked every 10 days, for a total of 14 harvests. At each harvest, the marketable yield per plant, i.e., net weight (kg) of green fruits of picked bunches, and the number of picked bunches per plant were recorded. Three representative harvests carried out on 88 DAT (II harvest), 125 DAT (VI harvest) and 188 DAT (XI harvest) were selected in order to match the beginning, middle and end of the harvesting period, respectively; the picked bunches were analyzed to measure a set of yield-related traits and the fruits from the proximal, central and distal portions of each bunch were grouped and separately analyzed to measure their qualitative characteristics, and bioactive compound and organic acid contents.

### 2.2. Yield-Related Traits and Qualitative Characteristics of Fruits

At each of the three selected harvests, the number of picked bunches per plant were first counted; then, on both the marketable and the unmarketable part of each bunch, the length (cm), weight (g) and fruit number were recorded. Moreover, on marketable fruits, a set of non-destructive and destructive quality analyses were performed.

The skin color of four fruits from the proximal, central and distal portion of each bunch were analyzed as CIELAB coordinates (i.e., L*, a* and b*). These were measured on four randomly selected surfaces of each fruit using a CM-700d spectrophotometer (Minolta Camera Co. Ltd., Osaka, Japan) with an 8 mm aperture diameter and calibrated with a Minolta standard white plate. L* refers to lightness (ranges from 0 (black) to 100 (white)); a* is the ratio of red and green colors (ranges from −60 (green) to +60 (red)); and b* is the ratio of yellow and blue colors (ranges from −60 (blue) to +60 (yellow)). Then, the a*/b* ratio, Chroma (C * = (a*2 + b*2)1/2) and hue angle (h° = arctan (b*/a*) values were calculated. The a*/b* ratio is an index that describes the color changes in tomato fruit [37,38]; C* represents color saturation, which varies from dull (low values) to vivid (high values); and h° is defined as a color wheel, with red-purple at an angle of 0°, yellow at 90°, bluish-green at 180° and blue at 270°. On the same fruits, the equatorial (DE, mm) and polar (DP, mm) diameters and dry matter (DM, %) after sample drying to a constant weight at 65 °C were determined. Four more marketable fruits from each bunch portion were analyzed to measure their soluble solid content (SSC), pH and titratable acidity (TA) [39,40]. The SSC, pH and TA were determined from the filtered juices of homogenized fruit tissues. In particular, the SSC was measured using a digital refractometer (model DBR35, Bioscientifica, Rome, Italy) and was expressed as °Brix; the pH was measured using a digital pH meter (GLP 22 pH meter, Crison Instruments, Barcelona, Spain); and the TA was measured by titration with 0.1 N NaOH up to pH 7.0 and was expressed as g citric acid 100 mL^−1^ of fresh juice.

### 2.3. Functional Properties of Fruits

At the three selected harvests, two more marketable fruits from the three bunch portions were analyzed to measure their functional properties, including the total polyphenol content (TPC), free radical-scavenging activity by the DPPH assay (DPPH), and the organic acid, lycopene and β-carotene contents.

The fruits were extracted according to the method reported by Luthria et al. [41] with modifications. Specifically, the samples were first ground protected from light and approximatively 400 mg of ground tissues was extracted with 10 mL of MeOH, in a sonicator bath (CP104 EIA) for 30 min at room temperature. The mixture was centrifuged at 6500× *g* for 15 min and the supernatant was transferred into a 20 mL flask. The residue was resuspended in 10 mL of MeOH, sonicated and centrifuged again. The supernatant was added to the previously collected supernatant and the volume was brought up to 20 mL with extraction solvent. The methanolic extract was stored at −20 °C until analysis. The TPC of the methanolic extract was measured by the Folin–Ciocalteu method, as described by Fan et al. [42]. Each sample was analyzed in triplicate. Gallic acid was used as a calibration standard to calculate the calibration curve at different concentrations (5–500 ppm). The results were expressed as mg of gallic acid equivalents (GAE) per 100 g of fw. The DPPH assay was carried out on the methanolic extract using the 2,2-diphenyl-1-picryl-hydrazyl (DPPH) method according to the procedure described by Fan et al. [42]. The determination was performed in triplicate for each sample. Trolox was used as a calibration standard to calculate the calibration curve at different concentrations (5–500 ppm) and the results were expressed as μmol of TEAC (Trolox Equivalent Antioxidant Capacity) per 100 g of fw. A liquid chromatograph Agilent 1200 Series system (Santa Clara, CA, USA) equipped with a Zorbax SB-C18 RRHT column (4.6 × 10 mm, 1.8 μm Agilent Technologies, Santa Clara, CA, USA) at 25 °C was used to separate and quantify the organic acids of the tomato fruits. Before the analysis, 2 mL of the methanolic extract of each sample was dried under a vacuum (SpeedVac Concentrator Savant SPD1010, Thermo Fisher Scientific Inc., Segrate (MI), Italy) and resuspended in 1 mL of mobile phase. Each sample (50 μL) was injected into the column and the separation was monitored at 214 nm for 30 min. The mobile phase was 0.1% (*v*/*v*) phosphoric acid in ultrapure water (HPLC-grade) with a flow rate of 0.5 mL/min. Malic acid, ascorbic acid, lactic acid, acetic acid, citric acid, succinic acid and fumaric acid were used as standards and the calibration curve was obtained from selected concentrations. The chromatographic peaks of the samples were identified according to the retention times of the standards. In the calculation of the organic acid amounts, the dilution of the samples was taken into account.

For the lycopene and β-carotene content determination, the fruits were extracted according to the method reported by Luengo et al. [43] with some modifications. Approximately 3 g of frozen and blended samples were weighed and 25 mL of the extracting solution, composed of hexane and ethanol (50:50, *v*/*v*), were added. The mixture was first stirred for 20 min, then sonicated for 10 min on ice and finally centrifuged at 6500× *g* for 15 min. The organic part was recovered and filtered with 0.45 μm PTFE filters. A liquid chromatograph Agilent 1200 Series system (Santa Clara, CA, USA) equipped with a Gemini C18 column (5 μm, 250 × 4.6 mm, Phenomenex, Torrance, CA, USA) set at room temperature was used to separate and quantify the lycopene and β-carotene levels. The separations were carried out using a flow rate of 1 mL/min and the detection was set at 450 nm. All of the sample was filtered into a 2 mL vial using a 0.45 μm PTFE filter and an aliquot (20 μL) was injected into the column. Linear gradient separation was achieved using the mobile phases 100% (*v*/*v*) acetonitrile (A) and 50:50 (*v*/*v*) hexane/ethanol (B). The analyses lasted 20 min. Lycopene and β-carotene were used as standards and the calibration curves were obtained from selected concentrations to calculate the amount of these compounds in the samples.

### 2.4. Statistical Analysis

All of the experimental data were first checked for normality and homogeneity of variance by applying the Shapiro–Wilk (*p* ≤ 0.05) and Bartlett (*p* ≤ 0.05) tests, respectively.

The cumulative marketable yield per plant during the harvesting period was analyzed for each treatment by fitting a nonlinear model of rectangular hyperbola [44,45] to the experimental data. Before being processed, the data were logarithmically transformed (log10 X); then, the following equation was applied:

(1) $$Y=\frac{Y_{\max}}{\left[1+\frac{K\;}{\left(X-X_{p}\right)}\right]}$$

where Y is the response variable (cumulative marketable yield); Y_max_ is the asymptotic Y value (or maximum Y value); K is the initial slope of the curve (or rate constant that determines the steepness of the curve); X is the explanatory variable (harvesting period); and X_p_ is the X value at which the reproductive phase of the crop cycle starts.

Model fitting was performed using the iterative least squares estimation method. The iterative procedure converged to the optimal values of the Y and K model parameters, which were considered significantly different (*p* ≤ 0.05) among the treatments if their 95% confidence intervals did not overlap. All the other experimental data collected from the three experimental treatments were tested for differences using analysis of variance (ANOVA). The statistical significance of the differences between the means was analyzed by the Tukey’s honest significance difference post hoc test at the 5% probability level. Specifically, data related to total marketable yield and total number of bunches per plant were analyzed by one-way ANOVA to evaluate the effect of the factor rootstock (R; three levels: Tb, Su and Ka). Data related to the yield-related traits were analyzed by two-way ANOVA to evaluate the effect of the factors harvest (H; three levels: II, VI and XI), rootstock (R) and their interaction. Finally, data on the qualitative characteristics and functional properties of the fruits were analyzed by three-way ANOVA to examine the effect of the factors harvest (H), rootstock (R), bunch portion (P; three levels: proximal, central and distal) and their interactions.

All the statistical analyses were performed using the JMP software package, version 15 (SAS Institute Inc., Cary, NC, USA).

## 3. Results

### 3.1. Marketable Yield during and at the End of the Harvesting Period

**Figure 2:**
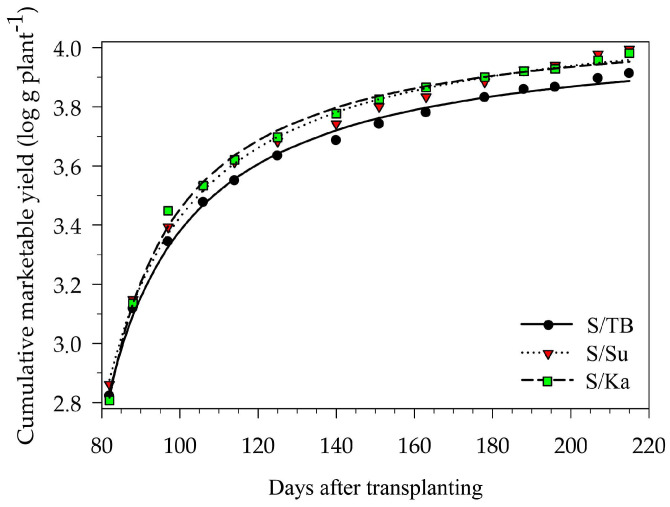
Cumulative marketable yield of the three considered scion/rootstock combinations, Sunstream/Top Bental (S/TB), Sunstream/Suzuka (S/Su) and Sunstream/Kaiser (S/Ka), during the harvesting period, from 82 to 215 days after transplanting. Points are the mean observed values and lines are the result of fitting the data to Equation (1).

Considering the results of the rectangular hyperbola model that was fitted to the yield data, three nonlinear regressions, which interpret the cumulative marketable yield per plant (log g plant^−1^) of each experimental treatment as a function of the harvesting period (DAT), were obtained (Figure 2). The estimates of the nonlinear model parameters (value ± standard error) and the summary of fit (R^2^, RMSE and number of observations) are reported in Table 2.

K, which represents the marketable yield per plant at the start of the harvesting period, was significantly higher when tomato plants were grafted onto the Suzuka (S/Su) than Kaiser rootstock (S/Ka), without any difference compared to Top Bental (S/TB, control). Y_max_, which represents the maximum marketable yield per plant at the end of the harvesting period, was significantly higher with both the S/Su and S/Ka treatments than with S/TB (control) (Figure 2 and Table 2).

The comparison of the estimated K and Y_max_ values among the three treatments indicate that the use of the Suzuka and Kaiser rootstocks increased the marketable yield per plant compared to the control. However, S/Su grafted plants produced more starting from the beginning of the harvesting period, confirming the very high vigor of the Suzuka rootstock (Table 2).

**Figure 3:**
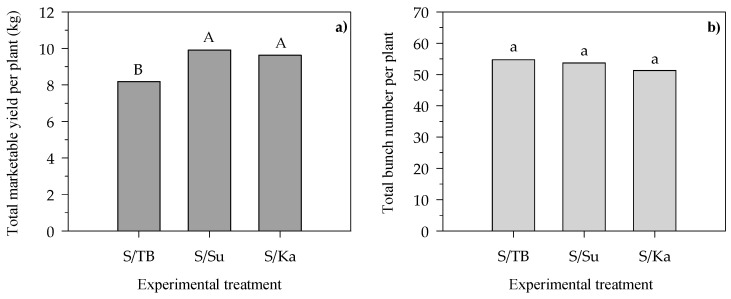
Total marketable yield per plant (**a**) and total bunch number per plant (**b**) of the three considered scion/rootstock combinations, Sunstream/Top Bental (S/TB), Sunstream/Suzuka (S/Su) and Sunstream/Kaiser (S/Ka), at the end of the harvesting period. Values are the means (*n* = 6). In each graph, different letters above the bars indicate significant differences among treatments (*p* ≤ 0.05, Tukey’s test). Uppercase letters refer to F test significance at *p* < 0.001.

The significant differences in the total marketable yield per plant among the treatments are more clearly showed in Figure 3, where the total number of picked bunches per plant are also displayed.

In comparison to S/TB (control), S/Su and S/Ka produced 20% higher (*p* < 0.001) total marketable yields (9.8 vs. 8.2 kg plant^−1^) (Figure 3a). Although the marketable yield per plant was higher in S/Su and S/Ka than in S/TB, the total number of picked bunches per plant (Figure 3b) did not differ among the three treatments, with an average value of 53.3 plant^−1^, as a consequence of the same number of productive stems maintained per plant in each treatment during the cultivation period (see Section 2.1).

### 3.2. Yield-Related Traits

**Figure 4:**
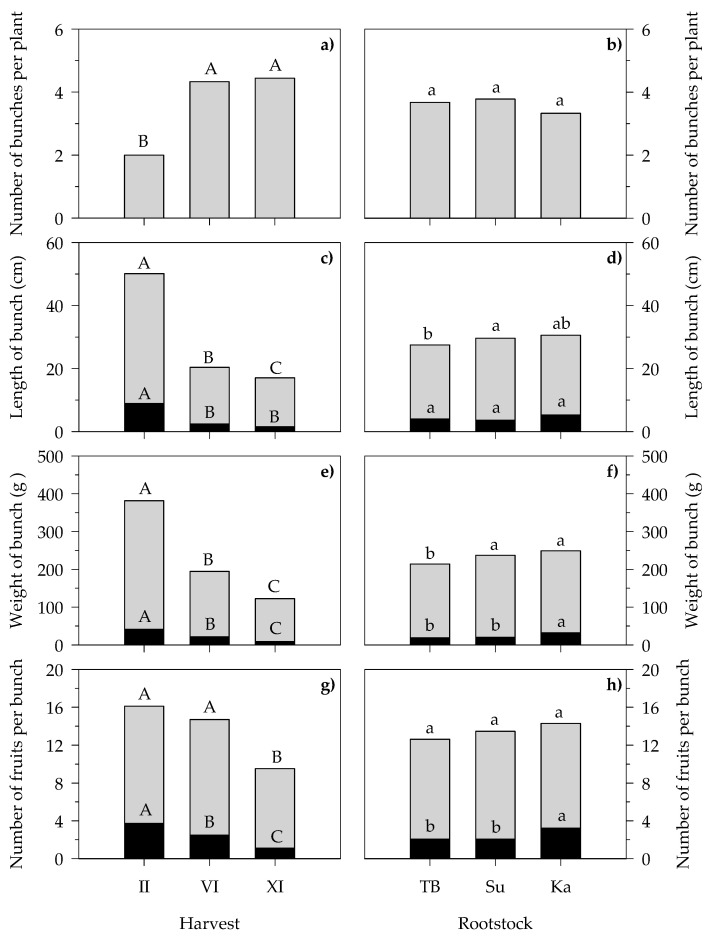
Main effects of harvest and rootstock on number of bunches per plant (**a**,**b**), length of bunch (**c**,**d**), weight of bunch (**e**,**f**) and number of fruits per bunch (**g**,**h**) of grafted cherry tomato plants. Grey and black bars indicate the marketable and unmarketable bunch parts, respectively. Values are the means (*n* = 6). Different letters above bars indicate significant differences among the means (*p* ≤ 0.05; Tukey’s test). Lowercase and uppercase letters refer to F test significance at *p* < 0.05 and 0.001, respectively. II, VI and XI harvests were carried out at 88, 125 and 188 days after transplanting, respectively. TB, Top Bental; Su, Suzuka; Ka, Kaiser.

The bunches produced by the S/TB, S/Su and S/Ka plants were analyzed further and their yield-related traits at the II, VI and XI harvests were characterized. The two-way ANOVA applied to the experimental data showed that the number of picked bunches per plant, as well as the yield-related traits of both the marketable and unmarketable bunch part, i.e., length, weight and number of fruits, were significantly affected by both of the considered experimental factors, harvest (H) and rootstock (R) (Figure 4). Except for the weight of the unmarketable part (data not shown), the interaction H × R did not show any significant influence on the considered parameters.

In terms of the harvest effect, the number of bunches per plant (Figure 4a) increased more than double passing from the II (2.0) to VI (4.3) and XI harvest (4.4), with the VI and XI harvest showing no difference. On the contrary, the length of both the marketable and unmarketable bunch parts (Figure 4c) significantly decreased passing from the II (41.2 and 8.9 cm, respectively) to VI (18.0 and 2.4 cm, respectively) and XI harvests (15.6 and 1.5 cm, respectively).

The weights of the marketable and unmarketable bunch parts (Figure 4e) also showed statistically significantly lower values passing from the II (340.4 and 41.3 g, respectively) to VI (175.2 and 21. 3 g, respectively) and XI harvests (113.0 and 9.2 g, respectively). The number of fruits per marketable bunch part (Figure 4g) was similar at both the II and VI harvests (on average, 12.3) and were significantly lowered by 31% at the XI harvest (8.4), while the number of fruits per unmarketable bunch part (Figure 4g) followed the same decreasing trend as the weight from the II (3.8) to VI (2.5) and XI harvests (1.1).

In terms of the rootstock effect, no significant differences in bunch number were detected among TB, Su and Ka grafted plants (on average, 3.6) (Figure 4b). Meanwhile, a significantly longer marketable bunch part was detected on plants grafted onto Su (26.1 cm) compared to TB (23.5) (Figure 4d) with no differences in the unmarketable part length among the TB, Su and Ka grafted plants. Moreover, both the Su and Ka grafted plants showed a higher weight for the marketable bunch part (on average, 216.7 g) than the TB grafted ones (195.0 g), while Ka showed the highest unmarketable part weight (32.4 g) compared to the TB and Su grafted plants that did not differ from each other (on average, 19.7 g) (Figure 4f). Finally, the highest number of fruits per unmarketable bunch part (Figure 4h) was observed on Ka grafted plants (3.2) and Su was not statistically different from TB (on average, 2.1). On the contrary, no significant differences were observed in the number of fruits per marketable bunch portion among plants grafted onto TB, Su and Ka rootstocks (on average, 11.0).

### 3.3. Qualitative Characteristics of Fruits

The qualitative characteristics of marketable tomato fruits were determined for each experimental treatment at the three selected harvests and with consideration of the three different bunch portions. The three-way ANOVA performed on the collected data showed a significant effect of the experimental factors harvest (H), rootstock (R) and bunch portion (P) (Table 3), as well as a significant influence of the two interactions H × R (Table 4) and H × P (Table 5). On the contrary, the interactions R × P and H × R × P did not significantly affected the considered parameters.

As reported in Table 3, L* reached the highest value at the XI harvest, while a* and b* were highest at the II and VI harvests. a*/b* and C * showed higher values at the II and VI harvests compared to the XI harvest. h° did not vary among the three harvests. The equatorial and polar diameters (D_E_ and D_P_) progressively decreased from the II to VI and XI harvests, while the dry matter content (DM) increased. The pH was highest at the II harvest, while the solid soluble content (SSC) showed a higher value at the II harvest than the VI one, which did not show any statistical difference from the XI harvest. The titratable acidity (TA) did not differ between the harvests.

The rootstock significantly influenced the skin color coordinates b* and C *, as well as the DM content, which were higher on fruits from TB and Ka compared to Su grafted plants. The D_E_ was also influenced by the rootstock, with higher values on fruits from Su and Ka grafted plants than TB ones. The other fruit qualitative characteristics did not vary among the three rootstocks (Table 3).

Moreover, fruits from the proximal and central bunch portions showed higher values of a*, a*/b*, C *, D_E_, DM content, SSC and pH than fruits from the distal bunch portion, which reached higher L* and TA values. Instead, fruits from the proximal bunch portion were characterized by the highest D_P_ value (Table 3). 

As reported above, the qualitative characteristics of fruits at the II, VI and XI harvests also varied depending on the rootstock (Table 4) and the bunch portion (Table 5).

The interaction between harvest and rootstock showed a significant effect only on the D_E_, D_P_, DM content and pH of the fruits, while the skin color coordinates, SSC and TA were not influenced. In particular, at the II harvest, the D_E_ and D_P_ of fruits from TP, Su and Ka grafted plants did not differ from each other, but were significant different from the values at both the VI and XI harvests (Table 4). The lowest D_E_ values were observed at the XI harvest on fruits from TB and Ka grafted plants (decreases of 21 and 23% compared to the II harvest). The lowest D_P_ values were detected at the VI harvest on TB grafted plant fruits and at the XI harvest on Ka ones (decreases of 20 and 21% compared to the II harvest, respectively). The DM content also varied among the three rootstocks, particularly at the VI and XI harvests, with Ka showing 8 and 17% higher values than Su, respectively, but there were no significant differences from the values of TB (Table 4). Finally, at the three harvests, the pH of fruits from Su and Ka grafted plants did not show any differences, while lower pH values were observed from TB fruits at both the VI and XI harvests than the II one (decreases of 23 and 22%, respectively).

Table 5 clearly shows that the interaction between harvest and bunch portion only had a significant effect on the skin color coordinates, L*, a* and C*, the two diameters D_E_ and D_P_, as well as on the pH of fruits. The other considered parameters, i.e., b*, a/b*, h°, DM, SSC and TA, were not influenced. More specifically, at the XI harvest, L* showed the highest value and a* and C* showed the lowest ones on fruits from the distal bunch portion. A lower D_E_, D_P_ and DM content were again observed from fruits from the distal bunch portion, particularly at the II harvest. Moreover, D_E_ did not differ among the three bunch portions at the VI harvest, while D_P_ and the DM content showed lower values in fruits from the distal bunch portion compared to the distal and central ones. D_P_ also varied among the fruits from the three bunch portions at the XI harvest, with lower values from fruits from both the central and distal portions compared to the proximal one. The pH of the fruits significantly varied among the three bunch portions at both the VI and XI harvests, with lower values from fruits from the distal portion.

### 3.4. Functional Properties of Fruits

**Figure 5:**
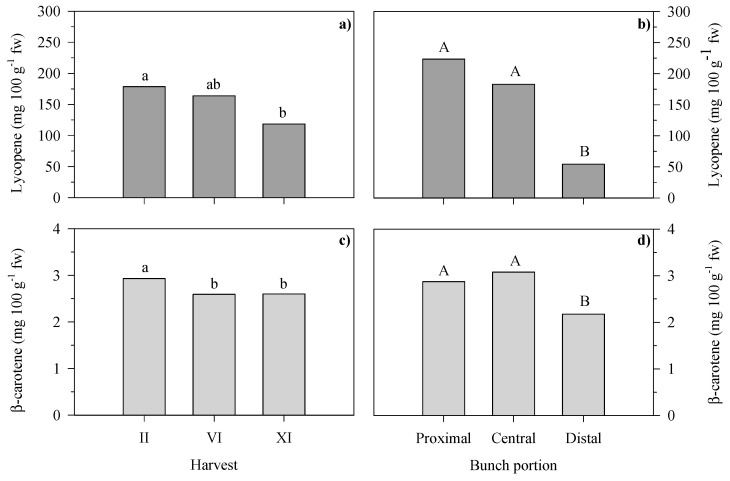
Main effects of harvest and bunch portion on lycopene (**a**,**b**) and β-carotene (**c**,**d**) contents of cherry tomato fruits. Values are the means (*n* > 6). Different letters above bars indicate significant differences among the means (*p* ≤ 0.05; Tukey’s test). Lowercase and uppercase letters refer to F test significance at *p* < 0.05 and 0.001, respectively. II, VI and XI harvests were carried out at 88, 125 and 188 days after transplanting, respectively.

The results of the three-way ANOVA performed on the data on the bioactive compound and organic acid contents of the cherry tomato fruits for each experimental treatment at the three harvests and on each bunch portion are reported in Table 6 and Figure 5.

The factor harvest (H) only had a significant effect on the total polyphenol content (TPC), the DPPH free radical-scavenging activity (DPPH) and the organic acid content (OA) (Table 6). In particular, fruits from the II harvest were characterized by the lowest TPC (Table 6) that increased by 11 and 15% at the VI and the XI harvest, respectively. On the contrary, at the II harvest, the fruits showed the highest DPPH activity (Table 6), which drastically decreased at the VI and XI harvests, with 71 and 87% lower values, respectively.

Regarding the OA content of the cherry tomato fruits (Table 6), no statistical difference were observed among the three selected harvests, with malic, ascorbic, citric and succinic acid contents showing, on average, values of 1.4, 0.03, 1.4 and 8.9 mg g^−1^ fw, respectively. On the contrary, the lactic and acetic acid contents were the highest at the II harvest, with no statistically significant differences between the VI and XI harvests (on average, 1.2 and 1.1 mg g^−1^ fw, respectively), accounting for 57 and 106% increases, respectively. Finally, the fumaric acid content showed a higher value at the II harvest and decreased by 50 and 100% at the VI and XI harvests, respectively.

Significant effects of both harvest (H) and bunch portion (P) factors on the lycopene and β-carotene contents of the tomato fruits were detected (Figure 5). The factor rootstock (R) and all the interactions (H × R, H × P, R × P and H × R × P) did not significantly influence the bioactive compound content of the cherry tomatoes.

In particular, the lycopene content (Figure 5a) was higher in tomato fruits from the II compared to the XI harvest, with a 51% increase (178.6 vs. 118.5 mg 100 g^−1^ fw); there was no statistically significant difference between the II and VI harvests (on average, 171.1 mg 100 g^−1^ fw). Likewise, a 13% higher β-carotene content (Figure 4c) was observed in fruits from the II harvest (2.9 mg 100 g^−1^ fw) compared to the VI and XI ones, which did not differ from each other (on average, 2.6 mg 100 g^−1^ fw).

Tomato fruits from the proximal and central portions were characterized by the highest content of lycopene (Figure 5b) (on average, 203.1 mg 100 g^−1^ fw) that decreases by 73% in the distal fruits. Similarly to lycopene, the β-carotene content (Figure 4d) was the highest in fruits from the proximal and central bunch portions, which were not statistically different from each other (on average, 3.0 mg 100 g^−1^ fw); the distal bunch portion had a β-carotene content of 2.2. g 100 g^−1^ fw, a 37% lower value.

## 4. Discussion

### 4.1. Yield-Related Traits

The findings of the present study highlighted that the use of Suzuka and Kaiser rootstocks positively influenced the marketable yield of grafted cherry tomato, showing this to be the better option to enhance the productive response of plants. This result suggests that both the Suzuka and Kaiser rootstocks were better adapted to the greenhouse micro-climatic conditions and responded better to the adopted cultivation practice than Top Bental, fully expressing their productive potential. Several authors have suggested that grafting tomato plants onto vigorous rootstocks improves fruit yield [46,47,48]. An increased efficiency of nutrient and water use, as a result of more vigorous root system development [49,50], was reported to play an important role in promoting the yield of grafted plants. Moreover, increases in the photosynthetic rate and modification of the endogenous plant hormone status by the rootstock were also suggested [51]. The significant differences in yield-related traits of bunches among the three considered rootstocks, particularly the length and weight, evidenced that Suzuka and Kaiser outperformed Top Bental, which is consistent with previous findings that reported the ability of several vigorous rootstocks to improve the performance of grafted plants [51,52,53].

Among the yield-related traits considered in the present study, the number, length and weight of bunches also showed variability depending on the harvesting time. In particular, an increase in bunch number and decreases in their length and weight were observed in the VI compared to the II harvest. However, this result was mainly linked to an increase in the density of productive stems from three (at the II harvest) to four (at the VI harvest) per plant, as a consequence of the cultivation practice adopted by the farmer hosting the experiment (see Section 2.1). In turn, the increase in productive stem density increased the number of bunches per plant; however, this stimulated the competition for living resources (water, nutrients, solar radiation and space) among the bunches themselves, which resulted in a shorter length and lower weight. These results are in agreement with those of other authors [52] who reported a significant influence of plant density on tomato plant biometric parameters and yield, as well as on fruit quality. The further decrease in the length and weight of bunches, as well as number of fruits per bunch at the XI harvest was due to plant senescence.

### 4.2. Qualitative Properties of Cherry Tomatoes

Regarding the qualitative characteristics of cherry tomato fruits, our results highlighted the significant effect of the harvesting time on almost all the considered parameters (except for the hue angle among the skin color coordinates, and the titratable acidity), which is consistent with previous findings [36,54]. In particular, tomatoes picked at the II harvest had the highest equatorial and polar diameters, as well as a higher solid soluble content and pH value, highlighting a larger fruit size and leading to a more intense flavor of the tomatoes. Moreover, the dry matter content of the fruits progressively increased from the II to the VI and XI harvests, consistent with the results reported from cherry tomato by other authors [26,36] who hypothesized that the higher temperatures occurring in the summer period increased transpiration and reduced the fruit water content, thus indirectly reducing the dry matter. In addition, the colorimetric analysis reported a better a*/b* ratio for fruits from the II harvest, indicating a redder fruit color, which was likely due to the better environmental conditions (temperature and solar radiation) for the biosynthesis of lycopene and β-carotene, the colored pigments responsible for the fruits’ red color [37]. The rootstock effects were less evident because only the equatorial diameter, dry matter content, and b* and C* values (among the skin color coordinates) showed significant differences. However, the higher equatorial diameter of tomato fruits from plants grafted onto Suzuka and Kaiser compared to that of Top Bental was further evidence of the higher yield performance of these two rootstocks. On the contrary, fruits from the three portions of the bunch (i.e., proximal, central and distal) showed large differences for the studied parameters, with the best performances in terms of size (equatorial and polar diameters), flavor (dry matter and solid soluble content, pH) and color (a*/b* and C* values) observed from fruits from the proximal bunch portion. These results could be explained by the fact that the ripening of fruits follows a gradient from the proximal to the distal portion of the bunch, as reported by a previous study [55]. The results are also in accordance with the findings of other authors [56] who reported that fruits from the proximal bunch section developed and assimilated photosynthates earlier and for a longer time compared to the distal ones.

### 4.3. Functional Properties of Cherry Tomatoes

The current literature suggests that the consumption of polyphenol-rich diets protects against several human degenerative diseases, including cancers, cardiovascular disease, type 2 diabetes, neurodegenerative diseases and aging by preventing the oxidative stress caused by free radicals, particularly ROS [57,58].

Phenolic compounds are considered as the primary antioxidants of tomato and other important classes of compounds with free radical-scavenger activity are carotenoids, especially lycopene, vitamin C and tocopherols [19]. The results of the present study showed a significant decrease in fruit antioxidant activity over the harvest period, despite the increase in the total polyphenol content. This was likely due to the significant reduction in lycopene, which accounts for more than 80% of the total carotenoid content in ripe tomato fruits and has the most powerful antioxidant activity among all the bioactive compounds and the highest quenching rate against singlet oxygen [20]. In turn, the increase in the total polyphenol content as well as the decrease in lycopene content in cherry tomato fruits as the cultivation period proceeded could be explained by the influence of environmental factors during the cultivation period. Rosales et al. [26] reported that in cherry tomato fruits cv. Naomi grown under two greenhouse types (parral and multispan), stress conditions, such as high temperatures, solar radiation and VPD, increased the total phenol, flavonoid and anthocyanin contents of fruits, as a mechanism of physiological acclimation to stress that improved the fruit quality. Indeed, the high temperatures of summer months are usually accompanied by intense solar radiation, inducing the accumulation of phenolic compounds such as flavonoids in the exocarp of fruits [59] since these compounds can absorb light or reduce sunscald and oxidative damage [26]. This likely occurred in our experiment where the increase in the total polyphenol content of tomato fruits at the VI and XI harvests could represent the physiological response of plants to the high temperature and solar radiation conditions. Similar results from cherry tomato were reported by other authors [26,36] who attributed the high total phenol content in fruits harvested in the summer months to the higher temperatures and sunshine that prevail during this period. It has also been reported that lycopene synthesis is inhibited at high temperatures (beyond 30–35 °C) and excessive solar radiation and β-carotene degradation is intensified at high temperatures (35–40 °C) [26,36]. The results of the present study (the decrease in the lycopene content over the cultivation period and the very low β-carotene content at the XI harvest) are consistent with these findings. The high solar radiation and temperature that occurred in the greenhouse during the summer period likely hindered lycopene synthesis and favored β-carotene degradation in the exocarp of the cherry tomato fruits. The present results are also consistent with those of previous studies [46,53] that showed that the nutritional quality of greenhouse grafted tomatoes was more affected by the harvest date than the rootstock type, indicating that the assessment of fruit quality needs to include various harvests in order to verify the effects of the environmental factors.

Regarding the effect of bunch portion on the bioactive compound content, tomatoes from the proximal and central portions were characterized by the highest lycopene and β-carotene contents in comparison to the distal fruits. These results indicate the different physiological maturity and ripening stages of tomato fruits along the bunch; the same trend was also observed in the a*/b* colorimetric coordinate and pH values, as well as in the soluble solid content (see Table 3), which is consistent with the findings of previous studies [36,37]. Indeed, lycopene and β-carotene, which are responsible for the red and the orange colors, respectively, accumulate during the ripening of tomato fruit in chloroplasts, which are progressively transformed into chromoplasts as the chlorophyll is degraded or metabolized [60], following a ripening gradient from the proximal to the distal portion of the bunch.

The organic acids (mainly citric and malic) and sugars (mainly glucose, fructose and sucrose)are the key taste compounds of cherry tomatoes. A high sugar concentration and relatively high acid content are required for optimal fruit flavor. In the present study, the fruit malic, ascorbic, citric and succinic acid contents did not vary between the different harvest times, thus indicating a similar fruit quality. In particular, malic, citric and succinic acids were the three major organic acids found in tomatoes, consistent with the findings of a previous study [61]. They are very important to the sourness of tomatoes because citric acid is characterized by a “burst” of tartness, malic acid has a smooth tartness, and succinic acid has a note of bitterness [30]. The malic, citric and succinic acid contents were within the ranges reported by other authors [26,61].

## 5. Conclusions

The yield traits, fruit qualitative characteristics and functional properties of cherry tomato, cultivar Sunstream, grafted onto three different rootstocks, i.e., Top-Bental, Suzuka and Kaiser, and grown in a greenhouse in a hydroponic culture were evaluated at three selected harvests (at the beginning, middle and end of the whole harvesting period) and from three bunch portions (proximal, central and distal).

The Kaiser and Suzuka rootstocks were shown to be the most promising as they induced a 20% higher marketable fruit yield in grafted plants than Top Bental, although they did not show any impact on the considered qualitative and functional properties of the fruit. These properties showed a high variability, indicating the considerable influence of both the harvesting time and the bunch portion. Similar to the fruits from the beginning of the harvesting period, tomatoes picked from the proximal and central bunch portions were characterized by a larger size (higher equatorial and polar diameters), better qualitative characteristics (higher solid soluble content and pH that likely lead a more intense flavor), a redder and more intense skin color (higher a*/b* ratio and C* values) and a higher bioactive compound content (lycopene and β-carotene).

The findings of this study, especially those related to the quality and the health-promoting compound content of fruits, could be useful and valuable information for growers and retailers, highlighting the factors that influence the production of cherry tomatoes with important nutritional and health properties and can be used to increase the market value of the product. This information could also be used to promote the functional role of this vegetable food product in the context of a healthy diet to consumers.

## Figures and Tables

**Table 1:** Characteristics of rootstock varieties tested in the experiment.

Rootstock Variety	Vigor	High Resistance	Intermediate Resistance
Top Bental F_1_	High	ToMV:0-2/Vd:0/Va:0/Fol:0-1/For/Pl/Sl	Mi/Ma/Mj ^1^
Suzuka RZ F_1_ (60-081)	Very high	ToMV:0-2/Fol:0,1/For/Pl/Va:0/Vd:0	Ma/Mi/Mj ^2^
Kaiser RZ F_1_ (61-077)	High	ToMV:0-2/Fol:0,1/For/Pl/Va:0/Vd:0	Ma/Mi/Mj ^3^

Fol, Fusarium oxysporum f.sp. lycopersici (fysio 0, 1); For, Fusarium oxysporum f.sp. radicis-lycopersici; Ma, Meloidogyne arenaria; Mi: Meloidogyne incognita; Mj, Meloidogyne javanica; Pl, Pyrenochaeta lycopersici; Sl, Stemphylium lycopersici; ToMV:0-2, tomato mosaic virus (strain 0, 2); Va, Verticillium albo-atrum (fysio 0); Vd, Verticillium dahliae (fysio 0). ^1^, https://tsiitalia.com/prodotto/top-bental/; ^2^, https://www.rijkzwaan.co.uk/vegetable-seeds/rootstock/suzuka-rz-f1-prdSL11203-ctgCrops.rootstock; ^3^, https://www.rijkzwaan.co.uk/vegetable-seeds/rootstock/kaiser-rz-f1-prdSL11085-ctgCrops.rootstock (accessed on 28 February 2024).

**Table 2:** Parameter estimates of the rectangular hyperbola model predicting the cumulative marketable yield (log g plant^−1^) of the three considered scion/rootstock combinations, Sunstream/Top Bental (S/TB), Sunstream/Suzuka (S/Su) and Sunstream/Kaiser (S/Ka), during the harvesting period, from 82 to 215 days after transplanting.

Experimental Treatment	K (log g Plant^−1^ Day^−1^)			Y_max_ (log g Plant^−1^)			Summary of Fit		
	Estimate	s.e.	95% Cl	Estimate	s.e.	95% CI	R^2^	RMSE	n. obs.
S/TB	6.88	0.17	6.54–7.23	4.07	0.01	4.04–4.09	0.968	0.06	84
S/Su	7.30	0.17	6.97–7.65	4.15	0.01	4.13–4.18	0.973	0.05	84
S/Ka	5.84	0.13	5.58–6.10	4.11	0.01	4.09–4.13	0.976	0.05	84

s.e., standard error; CI, confidence interval; n. obs., number of observations.

**Table 3:** Effect of harvest, rootstock and bunch portion on skin color coordinates (L*, a*, b*, a*/b*, C * and h°), equatorial (D_E_; cm) and polar (D_P_; cm) diameters, dry matter content (DM; %), solid soluble content (SSC; °Brix), pH and titratable acidity (TA; g citric acid 100 mL^−1^ fresh juice) of cherry tomato fruits.

Experimental Factor	L*	a*	b*	a*/b*	C *	h°	D_E_	D_P_	DM	SSC	pH	TA
**Harvest**												
II	28.88 b	29.08 a	41.77 a	0.70 ab	50.95 b	55.19 a	32.38 a	38.75 a	6.53 c	7.58 a	4.62 a	4.25 a
VI	29.95 b	32.13 a	42.84 a	0.75 a	53.74 a	53.39 a	27.29 b	31.53 c	6.81 b	6.82 b	4.16 b	4.19 a
XI	36.02 a	23.02 b	36.44 b	0.67 b	44.14 c	52.60 a	25.92 c	32.43 b	7.26 a	7.22 ab	4.16 b	4.39 a
*Significance*	***	***	***	*	***	ns	***	***	***	**	**	ns
**Rootstock**												
TB	32.21 a	27.63 a	40.61 a	0.70 a	49.63 ab	56.51 a	27.81 b	34.01 a	7.00 a	7.33 a	4.39 a	4.38 a
Su	32.54 a	26.23 a	38.10 b	0.71 a	47.02 b	49.03 a	29.20 a	34.68 a	6.53 b	7.10 a	4.19 a	4.15 a
Ka	31.52 a	29.85 a	41.60 a	0.72 a	51.48 a	54.88 a	28.57 a	34.02 a	7.07 a	6.99 a	4.21 a	4.32 a
*Significance*	ns	ns	**	ns	*	ns	***	ns	***	ns	ns	ns
**Bunch portion**												
Proximal	29.49 b	32.24 a	38.75 a	0.77 a	50.46 a	50.18 a	28.99 a	36.69 a	7.12 a	7.62 a	4.54 a	3.62 b
Central	30.59 b	29.63 a	40.66 a	0.72 a	50.38 a	53.98 a	29.04 a	34.47 b	7.00 a	7.33 a	4.23 a	4.13 ab
Distal	36.63 a	21.35 b	40.74 a	0.63 b	47.00 b	56.11 a	27.56 b	31.56 c	6.47 b	6.41 b	4.03 b	5.13 a
*Significance*	***	***	ns	**	*	ns	***	***	***	***	***	*

Values are the means (*n* > 6). In each column, means followed by different letters are significantly different (*p* ≤ 0.05; Tukey’s test). *, ** and *** indicate F test significance at *p* < 0.05, 0.01 and 0.001, respectively; ns, not significant. II, VI and XI harvests were carried out at 88, 125 and 188 days after transplanting, respectively. TB, Top Bental; Su, Suzuka; Ka, Kaiser.

**Table 4:** Effect of harvest x rootstock interaction on equatorial (D_E_, cm) and polar (D_P_, cm) diameters, dry matter content (DM, %) and pH of cherry tomato fruits.

Parameter	Harvest									*Significance*
	II			VI			XI			
	Rootstock			Rootstock			Rootstock			
	TB	Su	Ka	TB	Su	Ka	TB	Su	Ka	
**D_E_**	31.65 a	32.74 a	32.74 a	26.68 bc	27.51 b	27.68 b	25.11 d	27.34 b	25.31 cd	**
**D_P_**	38.21 a	38.88 a	39.16 a	30.74 c	31.95 bc	31.90 bc	33.07 b	33.21 b	31.01 c	*
**DM**	6.72 cd	6.46 d	6.42 d	6.76 cd	6.57 d	7.09 bc	7.51 ab	6.57 d	7.69 a	***
**pH**	5.34 a	4.33 ab	4.20 ab	4.11 b	4.20 ab	4.18 ab	4.14 b	4.11 b	4.24 ab	*

Values are the mean (*n* > 6). In each row, means followed by different letters are significantly different (*p* ≤ 0.05; Tukey’s test). *, ** and *** indicate F test significance at *p* < 0.05, 0.01 and 0.001, respectively. II, VI and XI harvests were carried out at 88, 125 and 188 days after transplanting, respectively. TB, Top Bental; Su, Suzuka; Ka, Kaiser.

**Table 5:** Effect of harvest x bunch portion interaction on skin color coordinates (L*, a* and C*), equatorial (D_E_; cm) and polar (D_P_; cm) diameters, dry matter content (DM; %) and pH of cherry tomato fruits.

Parameter	Harvest									*Significance*
	II			VI			XI			
	Bunch Portion			Bunch Portion			Bunch Portion			
	Proximal	Central	Distal	Proximal	Central	Distal	Proximal	Central	Distal	
L*	28.32 bc	28.67 c	30.07 bc	28.11 c	28.48 c	33.26 bc	31.25 bc	34.61 b	42.19 a	*
a*	30.11 a	30.07 a	25.08 a	33.83 a	32.42 a	30.15 a	31.37 a	26.38 a	11.31 b	***
C *	51.12 ab	51.90 ab	47.91 ab	53.42 a	53.13 a	54.67 a	47.27 b	46.13 b	39.02 c	**
**D_E_**	33.39 a	33.57 a	30.17 b	27.53 cd	27.57 e	26.78 cde	26.06 de	25.98 e	25.72 e	***
**D_P_**	42.56 a	39.52 b	34.17 c	33.22 cd	31.98 de	29.39 f	34.28 c	31.91 de	31.11 ef	***
**DM**	6.93 ab	6.74 bc	5.92 d	7.12 ab	6.92 ab	6.39 c	7.33 a	7.33 a	7.12 ab	**
**pH**	5.91 a	4.29 a	4.34 a	4.29 a	4.18 ab	4.02 bc	4.32 a	4.23 a	3.94 c	*

Values are the means (*n* > 6). In each row, means followed by different letters are significantly different (*p* ≤ 0.05; Tukey’s test). *, ** and *** indicate F test significance at *p* < 0.05, 0.01 and 0.001, respectively. II, VI and XI harvests were carried out at 88, 125 and 188 days after transplanting, respectively.

**Table 6:** Main effects of harvest on total polyphenol content (TPC, mg GAE 100 g^−1^ fw), free radical-scavenging activity (DPPH, μmol TEAC 100 g^−1^ fw) and organic acid content (mg g^−1^ fw) of cherry tomato fruits.

Experimental Factor	TPC	DPPH	Organic Acid Content						
			Malic	Ascorbic	Lactic	Acetic	Citric	Succinic	Fumaric
**Harvest**									
II	2.12 b	0.0028 a	1.392 a	0.032 a	1.822 a	2.317 a	4.854 a	8.848 a	0.009 a
VI	2.36 ab	0.0008 b	1.468 a	0.025 a	1.313 b	1.231 b	4.744 a	9.045 a	0.005 b
XI	2.43 a	0.0004 c	1.387 a	0.025 a	1.005 b	1.018 b	3.599 a	8.775 a	0.000 c
*Significance*	*	***	ns	ns	***	***	ns	ns	***

Values are the means (*n* > 6). In each column, means followed by different letters are significantly different (*p* ≤ 0.05; Tukey’s test). * and *** indicate F test significance at *p* < 0.05 and 0.001, respectively. ns, not significant.

## Data Availability

The original contributions presented in the study are included in the article, further inquiries can be directed to the corresponding author.
